# Poised chromatin and bivalent domains facilitate the mitosis-to-meiosis transition in the male germline

**DOI:** 10.1186/s12915-015-0159-8

**Published:** 2015-07-22

**Authors:** Ho-Su Sin, Andrey V. Kartashov, Kazuteru Hasegawa, Artem Barski, Satoshi H. Namekawa

**Affiliations:** Division of Reproductive Sciences, Division of Developmental Biology, Perinatal Institute, Cincinnati Children’s Hospital Medical Center, Cincinnati, OH 45229 USA; Division of Allergy and Immunology, Division of Human Genetics, Cincinnati Children’s Hospital Medical Center, Cincinnati, OH 45229 USA; Department of Pediatrics, University of Cincinnati College of Medicine, Cincinnati, OH 49229 USA; Present address: Department of Developmental Biology, Department of Genetics, Stanford University School of Medicine, Stanford, CA 94305 USA; Present address: Department of Medicine, Stanford University School of Medicine, Stanford, CA 94305 USA

**Keywords:** Germ cells, Epigenome, Sex chromosomes, Meiosis, Spermatogenesis

## Abstract

**Background:**

The male germline transcriptome changes dramatically during the mitosis-to-meiosis transition to activate late spermatogenesis genes and to transiently suppress genes commonly expressed in somatic lineages and spermatogenesis progenitor cells, termed somatic/progenitor genes.

**Results:**

These changes reflect epigenetic regulation. Induction of late spermatogenesis genes during spermatogenesis is facilitated by poised chromatin established in the stem cell phases of spermatogonia, whereas silencing of somatic/progenitor genes during meiosis and postmeiosis is associated with formation of bivalent domains which also allows the recovery of the somatic/progenitor program after fertilization. Importantly, during spermatogenesis mechanisms of epigenetic regulation on sex chromosomes are different from autosomes: X-linked somatic/progenitor genes are suppressed by meiotic sex chromosome inactivation without deposition of H3K27me3.

**Conclusions:**

Our results suggest that bivalent H3K27me3 and H3K4me2/3 domains are not limited to developmental promoters (which maintain bivalent domains that are silent throughout the reproductive cycle), but also underlie reversible silencing of somatic/progenitor genes during the mitosis-to-meiosis transition in late spermatogenesis.

**Electronic supplementary material:**

The online version of this article (doi:10.1186/s12915-015-0159-8) contains supplementary material, which is available to authorized users.

## Background

The germline is the only heritable lineage that ensures continuity of life. A unique feature of the germline is the plasticity of its epigenetic state, which is supported by cycles of programming and reprogramming that lead to acquisition of totipotency in the next generation [1, 2]. During embryonic development, primordial germ cells (PGCs) undergo extensive epigenetic reprogramming prior to the sexual differentiation of male and female germ cells [3, 4]. Male germ cells go through male-specific epigenetic programming during later stages of germ cell differentiation in adult testes, where they undergo self-renewal, enter meiosis, and differentiate into sperm. This male-specific differentiation accompanies massive cellular reconstruction and chromatin remodeling [5].

A common feature of male germ cells and embryonic stem (ES) cells is the suppression of developmental promoters with bivalent domains of H3K4me3 and H3K27me3. In ES cells, developmental regulator genes are silent but poised for activation at later developmental stages with bivalent domains [6]. In mature sperm, bivalent domains are also found on developmental promoters that are not expressed in early embryos but are activated later during development [7, 8]. These developmental genes are silent throughout germ cell development, and bivalent domains on these genes are found in PGCs [9–11] and adult germline stem (GS) cells [12], and are maintained into pachytene spermatocytes (PS) and postmeiotic round spermatids (RS) [11, 13]. Based on these findings, it is believed that bivalent domains maintain the development potential of these genes throughout the germline. However, how male germline-specific events are regulated during critical stages of spermatogenesis remains largely unknown.

In addition to epigenetic changes on autosomes, sex chromosomes undergo unique epigenetic programming during the meiotic and postmeiotic stages. In mammalian males, X and Y chromosomes are unsynapsed during meiosis in PS, and are specifically silenced by the action of DNA damage response (DDR) factors such as γH2AX and its binding partner MDC1 [14–16]. This chromosome-wide silencing is called meiotic sex chromosome inactivation (MSCI). Sex chromosome inactivation is maintained into postmeiotic RS in a distinct transcriptionally silent compartment termed postmeiotic sex chromatin (PMSC) [17–19]. In spite of postmeiotic silencing in round spermatids, a set of sex chromosome-linked genes, which function in late spermatogenesis, escapes postmeiotic silencing and is activated [17, 20]. This escape gene activation is controlled by the DDR factor RNF8, an interacting partner of MDC1 [21, 22]. Currently, the epigenetic landscape associated with epigenetic programming of the sex chromosomes during meiosis remains largely unknown.

In this study, we performed comprehensive epigenomic and transcriptomic profiling during the critical stages of spermatogenesis using chromatin immunoprecipitation sequencing (ChIP-seq) and RNA-seq, and demonstrate that global epigenetic changes underlie extensive transcriptional alterations during spermatogenesis. Our epigenomic analyses further clarify the distinct chromatin environments of autosomes and sex chromosomes during spermatogenesis, and reveals that poised chromatin and formation of bivalent domains underlie genome-wide epigenetic changes during late spermatogenesis.

## Results

### Genome-wide transcriptional change during the mitosis-to-meiosis transition

**Fig. 1:**
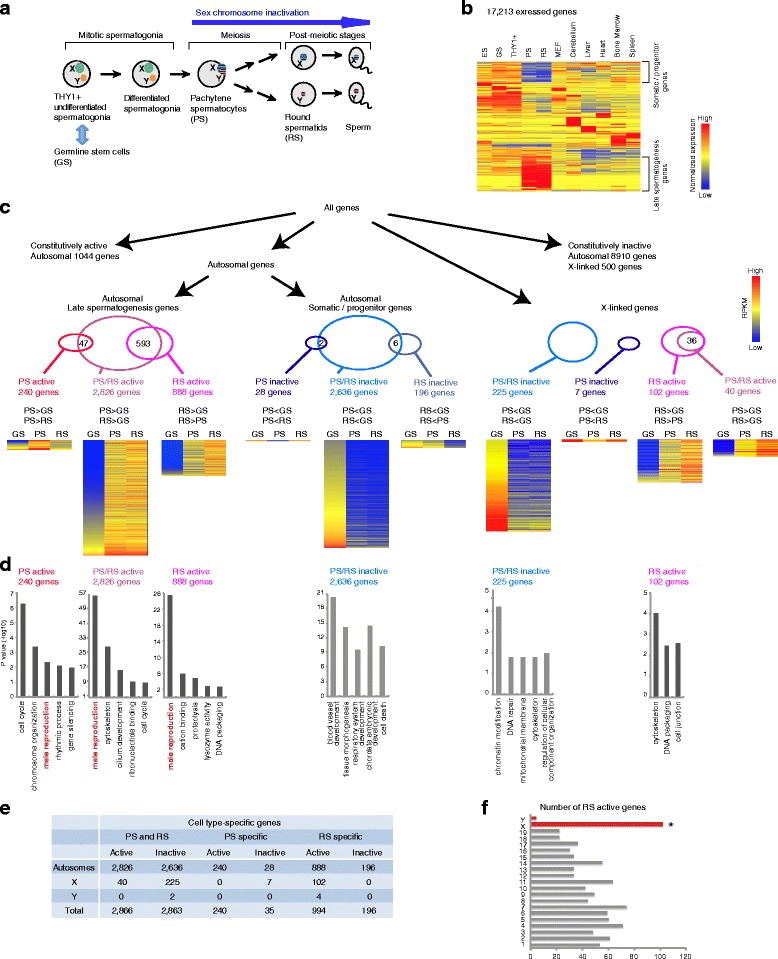
The global transcriptome changes during the late stages of male germline. **a** Schematic of spermatogenesis. In this study, germline stem (GS) cells were used as the representative stage of the stem cell phase. X chromosomes are depicted in green and the Y chromosomes are depicted in orange. Barred chromosomes represent suppressed transcription. **b** A heatmap showing gene expression patterns among several germ cells versus somatic cells. All 17,213 genes that showed more than 3 RPKM in at least one cell type are shown. RNA-seq data were obtained from published studies as described in the Methods section. **c** Flow chart of grouping of each class of spermatogenesis genes, degree of overlaps, and their expression heatmaps. **d** Gene ontology analysis of each class of spermatogenesis genes. **e** Summary table of each class of spermatogenesis genes. **f** Enrichment of RS active genes on the X chromosome. *P <2.2e-16, chi-square test. ES, embryonic stem cells; GS, germline stem cells; MEF, mouse embryonic fibroblasts; PS, pachytene spermatocytes; RPKM, reads per kilobase per million; RS, round spermatids; THY1+, THY1+ undifferentiated spermatogonia

To assess genome-wide gene expression changes during the mitosis-to-meiosis transition in spermatogenesis, we have recently performed RNA-seq analysis at three representative time points before, during, and after meiosis (Fig. 1a) [23]. Because purified spermatogonia consist of a heterogeneous cell population, it is difficult to obtain a large number of homogenous cells for ChIP-seq analysis. Because of this, we used cultured GS cells [24] as a representative stage of the mitotic phase of spermatogenesis. Our cultured GS cells exhibited a gene expression profile similar to THY1+ undifferentiated spermatogonia cells purified from mouse testes (Fig. 1b), confirming that the GS cells recapitulate undifferentiated spermatogonia *in vivo*. For meiotic and postmeiotic stages, we used purified PS and RS, respectively (Fig. 1a). To identify the unique features of germline transcriptomes during spermatogenesis, we compared RNA-seq data from these cell types to the published RNA-seq data obtained from THY1+ undifferentiated spermatogonia, ES cells, somatic cells, and tissues (see Methods section). A heatmap analysis of 17,213 genes expressed (reads per kilobase per million (RPKM) >3) in at least one condition revealed that a significant transcriptional change occurs during the mitosis-to-meiosis transition, and that the transcriptomes of PS and RS are largely different from that of the mitotic phase of spermatogenesis as well as other somatic cells and tissues (Fig. 1b). Two distinct features that are common in PS and RS transcriptomes are activation of late spermatogenesis genes, as previously described [17, 25, 26], and suppression of a large group of genes that are commonly expressed in the somatic phase and spermatogenesis progenitor cells. Herein we will refer to the latter group of genes as somatic/progenitor genes. This analysis suggests that there is a massive transcriptional change at the mitosis-to-meiosis transition during differentiation of mitotic spermatogonia into meiotic spermatocytes, and that transcriptomes during the late stages of spermatogenesis are significantly different from that of somatic lineages [23].

### Unique features of transcriptomes during meiosis and postmeiosis

To define lists of somatic/progenitor genes and late spermatogenesis genes, the transcriptomes were separated into gene groups according to the following criteria (Fig. 1c,d,e): (1) four-fold change in pairwise comparisons between GS and PS, GS and RS, or PS and RS; (2) adjusted *P* value ≤0.05 for significance of differential expression among the cell types; and (3) RPKM in at least one cell type ≥5. Because sex chromosomes are subject to unique epigenetic programming during meiosis and postmeiosis, we analyzed expression profiles of genes located on autosomes and sex chromosomes separately.

On autosomes, we found that 2,826 genes were commonly active in PS and RS (abbreviated as PS/RS active genes hereafter), and further defined the list of PS- or RS-specific active and inactive genes (abbreviated as PS active, PS inactive, RS active, RS inactive, respectively; Fig. 1c). Gene ontology (GO) enrichment analysis revealed that male reproduction-associated genes are significantly enriched in PS/RS active, PS active genes, and RS active genes (Fig. 1d). On the other hand, 2,636 autosomal genes were commonly inactive in PS and RS (abbreviated as PS/RS inactive genes hereafter; Fig. 1c). GO enrichment analysis reveals that the PS/RS inactive gene set is enriched with genes involved in somatic functions such as blood vessel development and tissue morphogenesis, suggesting that they are presumably dispensable during the late stages of spermatogenesis (Fig. 1d). We also identified 1,044 constitutively active genes and 8,910 constitutively inactive genes on autosomes in all three cell types (see Methods).

Because of the paucity of Y-linked genes, we focused on the X chromosome for detailed analysis. On the X chromosomes, 225 genes were significantly repressed in both PS and RS, consistent with sex chromosome inactivation (Fig. 1c), and the GO analysis demonstrated that this group of genes was highly associated with chromatin modification (Fig. 1d). On the other hand, 102 X-linked genes escaped sex chromosome inactivation and were predominantly expressed in RS. Interestingly, this group of genes is specifically expressed in the germline (Additional file 1: Figure S1), and is disproportionately enriched on the X chromosome (102/994, 10.3 %, *P* <2.2e-16, chi-square test) when compared with those on the autosomes (Fig. 1f). Additionally, we identified 500 X-linked constitutively inactive genes in all three cell types (see Methods). Taken together, our RNA-seq data are in accord with previous gene expression studies [12, 17, 21, 25–27], and these results confirm distinct regulation between autosomes and the X chromosome in spermatogenesis.

### Distinct epigenetic landscapes between autosomes and the X chromosome during spermatogenesis

To elucidate the epigenetic principles of mouse spermatogenesis, we performed ChIP-seq chromatin profiling in GS, PS, and RS cells. In particular, we examined the distribution of RNA polymerase II (RNAPII) and representative active epigenetic modifications such as H3K4me2, H3K4me3, H4K8ac, H4K16ac, and histone lysine crotonylation (Kcr). In addition to active modification, we examined representative silent modifications H3K27me3 and H3K9me2.

**Fig. 2:**
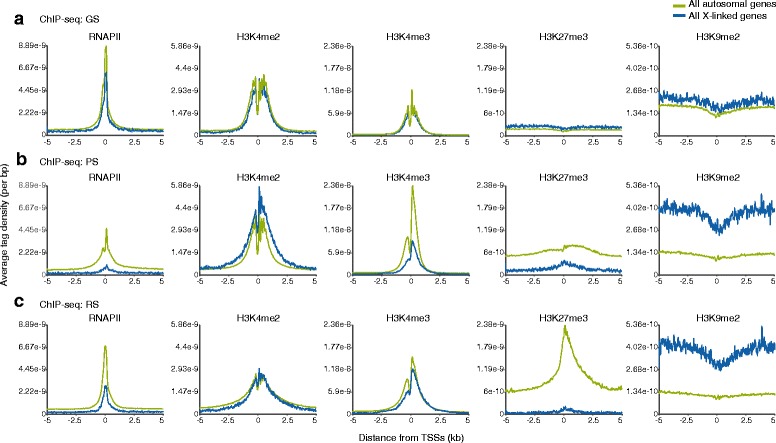
Distinct regulation between the X chromosome and autosomes during the late stages of male germline. **a** GS, **b** PS, and **c** RS are shown. Average tag density (ATD) of each histone mark was compared between all autosomal genes and all X-linked genes. ATD, average tag density; GS, germline stem cells; PS, pachytene spermatocytes; RS, round spermatids; ChIP-seq, chromatin immunoprecipitation sequencing

To account for the distinct regulation of gene expression from autosomes and the X chromosome during spermatogenesis, we first compared the average tag density (ATD) profiles of these modifications around transcription start sites (TSSs) between all autosomal genes and all X chromosome-linked genes during spermatogenesis (Fig. 2a,b,c). Based on the limited availability of annotated sequences on the Y chromosome, Y chromosome data were excluded from our analysis hereafter. Consistent with the phenomena of almost complete silencing in MSCI and postmeiotic RS-specific escape gene activation, RNAPII was largely depleted from the X chromosome in PS and slightly increased in RS (Fig. 2b,c). Although distribution of H3K4me2 and H3K4me3 was comparable between autosomes and X chromosome in GS (Fig. 2a), H3K4me2 was slightly enriched on the X chromosome in PS consistent with the cytological localization that H3K4me2 starts to accumulate on the sex chromosomes during the late pachytene stage [21, 28], whereas H3K4me3 was enriched on autosomes at this stage (Fig. 2b). H4K8ac, Kcr, and H4K16ac were also enriched on autosomes in PS, and, curiously, H4K16ac was continuously low on the X chromosomes compared to autosomes in all three cell types (Additional file 1: Figure S2). In contrast to these active modifications, H3K9me2 was slightly enriched on the X chromosome compared to autosomes in GS cells (Fig. 2a). In PS and RS, consistent with cytological localization [17], H3K27me3 was largely depleted from the X chromosome, whereas H3K9me2 was enriched there (Fig. 2b,c). In contrast, H3K27me3 accumulated on autosomes in PS and was highly enriched on autosomal TSSs in RS. Therefore, these results suggest that autosomes and the X chromosomes are subject to distinct modes of epigenetic regulation during spermatogenesis. Because of the distinct regulation, we proceeded to analyze the epigenomes of autosomes and sex chromosomes separately.

### Autosomal late spermatogenesis genes are silent, but poised in GS cells for later activation in PS

**Fig. 3:**
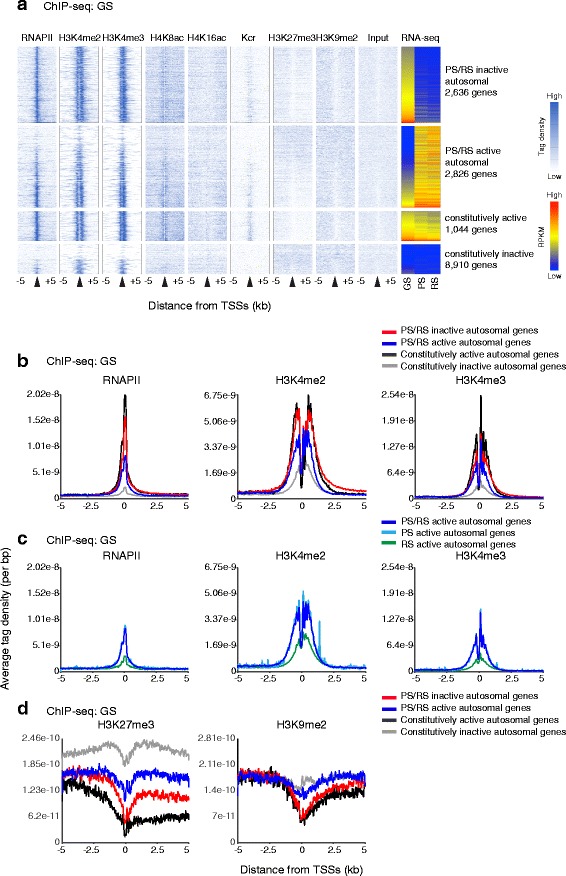
Autosomal late spermatogenesis genes are poised in GS cells for activation at PS. **a** A heatmap showing distribution of histone marks in GS cells. Tag density around TSS (±5 kb) is shown. **b** ATD of active marks in representative groups in GS cells. **c** ATD of active marks at the genes activated in later stages. **d** ATD of silent marks in representative groups in GS cells. ATD, average tag density; GS, germline stem cells; PS, pachytene spermatocytes; TSS, transcription start site

We first focused on the events on the autosomes and investigated whether the genes specifically regulated during spermatogenesis undergo epigenetic changes during differentiation. Heatmap analyses revealed that, in GS cells, H3K4me2 and H3K4me3 were highly accumulated on the active genes (both constitutively active genes and PS/RS inactive genes) (Fig. 3a). ATD analysis revealed that RNAPII and H3K4me3 were highly accumulated on TSSs of these genes, and that H3K4me2 localization is broader than localization of RNAPII and H3K4me3, and accumulated on the region surrounding TSSs of these genes (Fig. 3b). H4K8ac and Kcr were also accumulated around TSSs of constitutively active genes, but were less intense on the PS/RS inactive genes that are highly expressed in GS cells (Additional file 1: Figure S3), suggesting that, in GS cells, gene activation is distinctly regulated between PS/RS inactive genes and constitutively active genes. Consistent with this notion, H4K16ac accumulated on TSSs of constitutively active genes, but not on the TSSs of PS/RS inactive genes (Additional file 1: Figure S3).

Notably, RNAPII, H3K4me2, and H3K4me3 were largely present on PS/RS active genes even though these genes were silent in GS cells (Fig. 3a,b). Notably, ATD of these modifications on PS/RS active genes overlapped with that of PS active genes in GS cells, but RS active genes did not exhibit enrichment of active modifications in GS cells (Fig. 3c). Further, the H3K27me3 level of PS/RS active genes was lower than that of constitutively inactive genes, whereas H3K9me2 did not show this reduction (Fig. 3d). These results suggest that the autosomal genes activated in PS are already epigenetically poised by deposition of active modifications and RNAPII, as well as by reduction of H3K27me3, for future activation. Similar epigenetic gene poising was observed in T cells for genes that are inducible during T cell activation and in other systems [29, 30]. Taken together, we conclude that activation of autosomal late spermatogenesis genes in PS is preprogrammed in GS cells.

### H3K4me2 remained on somatic/progenitor genes after gene inactivation in PS

**Fig. 4:**
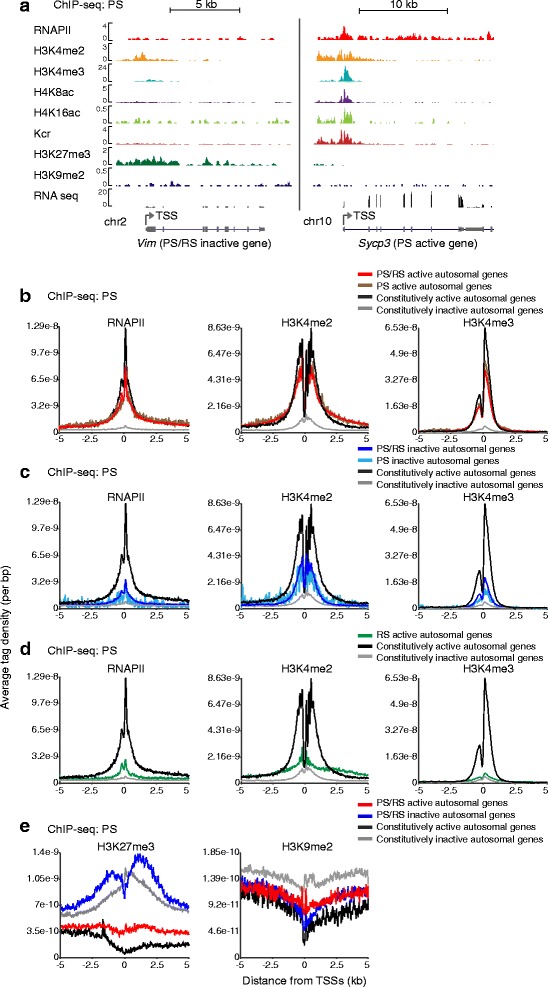
Active marks remain after the inactivation of autosomal somatic/progenitor genes in PS. **a** Distribution of histone marks around PS/RS inactive *Vim* gene locus and PS active *Sycp3* gene locus in PS. **b** ATD of active marks at the active genes in PS. **c** ATD of active marks at the silent genes in PS. **d** ATD of active marks in PS. These genes are activated in RS. **e** ATD of each silent mark in representative groups in PS. ATD, average tag density; PS, pachytene spermatocytes; RS, round spermatids

Next, we sought to examine how the meiosis-specific transcriptome is regulated for autosomes during the PS stage. At the PS active gene synaptonemal complex protein 3 (*Sycp3*), active modifications such as H3K4me3, H4K8ac, H4K16ac, and Kcr were highly accumulated at the TSS, and H3K4me2 exhibited a broader peak of enrichment near the TSS (Fig. 4a). These profiles of active modifications were common among PS/RS active genes, PS active genes, and constitutively active genes, suggesting that PS-specific gene activation is regulated by a similar epigenetic mechanism with that of constitutively active genes in PS (Fig. 4b, Additional file 1: Figure S4). On the other hand, genes inactivated in PS such as vimentin (*Vim*) exhibited a distinct feature compared to the constitutively inactive genes: H3K4me2 largely remained in PS at PS/RS inactive genes and PS inactive genes although RNAPII and H3K4me3 were largely depleted (Fig. 4a,c). In addition, the silent modification H3K27me3, but not H3K9me2, was highly enriched at PS/RS inactive genes (Fig. 4a,e). These results suggest that bivalent chromatin signatures such as H3K27me3 with H3K4me2 are associated with PS/RS inactive genes in PS.

We further investigated whether there is any epigenetic signature that predicts RS-specific gene activation in PS. Both H3K4me2 and Kcr were broadly enriched at RS active genes in PS, but H3K4me3 did not show enrichment compared to that of constitutively inactive genes (Fig. 4d, Additional file 1: Figure S4). Thus, on RS active genes, H3K4me2 and Kcr, but not H3K4me3, are already established in PS for future activation in RS.

### Unique epigenetic landscape of late spermatogenesis genes on autosomes in RS

**Fig. 5:**
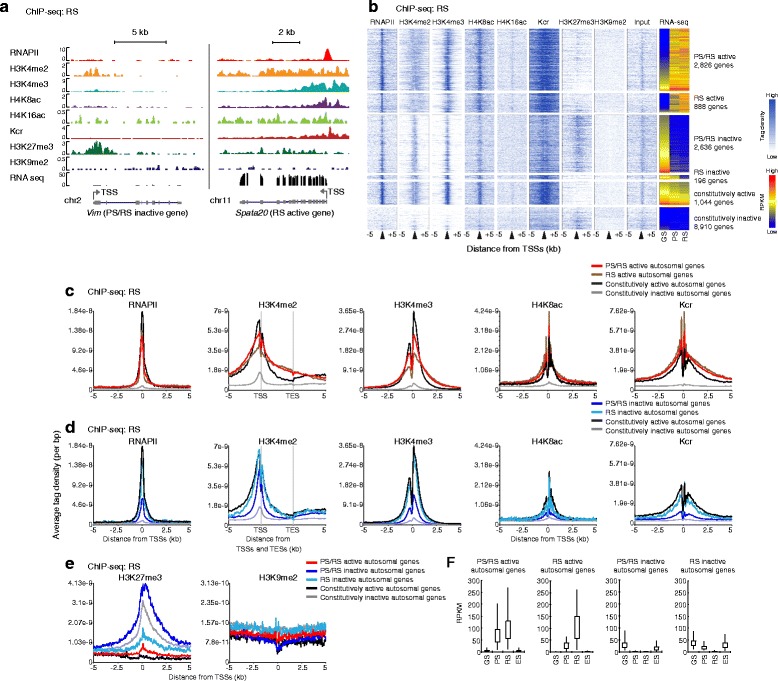
Epigenetic profiles for RS-specific activation and gene poising on autosomal somatic/progenitor genes in RS. **a** Distribution of histone marks on PS/RS inactive *Vim* gene locus and RS active *Spata20* gene locus in RS. **b** A heatmap showing distribution of histone marks in RS. Density around TSS (±5 kb) is shown. **c** ATD of active marks at the active genes in RS. For H3K4me2, ATD on gene bodies from TSS to TES and ±5 kb regions were analyzed. **d** ATD of active marks at the silent genes in RS. **e** ATD of silent marks in representative groups in RS. **f** Expression of each gene set in GS, PS, RS, and ES. ATD, average tag density; ES, embryonic stem cells; GS, germline stem cells; PS, pachytene spermatocytes; RS, round spermatids; TSS, transcription start site

We next investigated the epigenetic signature of autosomes in postmeiotic RS specifically focusing on the RS active genes. Interestingly, active epigenetic modifications were present not just at the promoters of the RS active genes, such as spermatogenesis-associated 20 (*Spata20*), but were spread out broadly from TSSs to transcription end sites (TESs) (Fig. 5a). In the heatmap and ATP analysis, H3K4me2 was highly enriched in the gene bodies but less enriched at the TSSs of RS active genes compared to constitutively active genes (Fig. 5b,c). Contrastingly, H3K4me2, H4K8ac, and Kcr were highly enriched on the TSSs of RS active genes even compared to constitutively active genes (Fig. 5c), while H4K16ac did not show such a difference (Additional file 1: Figure S5). These results reveal the unique epigenetic landscape of late spermatogenesis genes on autosomes in RS.

### Autosomal somatic/progenitor genes are silent in RS via the deposition of bivalent epigenetic marks

We next examined the epigenetic signature of somatic/progenitor genes inactivated in RS. H3K4me2 remained around the TSSs of PS/RS inactive genes such as *Vim* (Fig. 5a). RNAPII and H3K4me2/3 were present on both RS inactive genes and PS/RS inactive genes (Fig. 5b,d). Importantly, H3K27me3 was deposited at the TSS of PS/RS inactive genes in RS compared to PS/RS inactive genes in PS, while H3K9me2 did not exhibit this feature (Figs. 4e and 5a,e). This suggests that the deposition of bivalent marks at the TSS of somatic/progenitor genes occurred in the transition between PS and RS without expression changes. To determine whether somatic/progenitor genes are poised for activation after fertilization, we compared their gene expression during spermatogenesis and in ES cells, which represent the post-fertilization inner cell mass of blastocysts. Consistent with our global expression analysis (Fig. 1b), somatic/progenitor genes (RS and PS/RS inactive genes) are expressed in ES cells, whereas late spermatogenesis genes (PS/RS and RS active genes) are not expressed in ES cells (Fig. 5f). Therefore, these results suggest that the somatic/progenitor program is suppressed in late spermatogenesis, but poised for activation after fertilization. Importantly, in contrast to the class of bivalent domains on developmental promoters that are consistently silent throughout the male germline [11, 13], our analysis reveals a new class of bivalent genes that are expressed in ES and GS cells but are temporarily suppressed in PS and RS. This suggests a novel function of bivalent domains: suppression of the somatic/progenitor program during late spermatogenesis.

### X-linked genes subject to MSCI and postmeiotic silencing are poised for activation after fertilization without the formation of bivalent domains

Because sex chromosomes undergo MSCI and are regulated separately from autosomes in spermatogenesis (Fig. 2), we investigated the epigenetic landscape of the X chromosomes separately from that of autosomes. In Fig. 1, we classified X-linked genes into two major categories: the major group consists of 225 genes that are active in GS but are subject to both MSCI and postmeiotic silencing (PS/RS inactive); the other group consists of 102 genes that are not expressed in GS, but are highly activated in RS (RS active). This latter group is also referred to as escape genes because they escape chromosome-wide postmeiotic silencing in RS and are activated [20, 21].

**Fig. 6:**
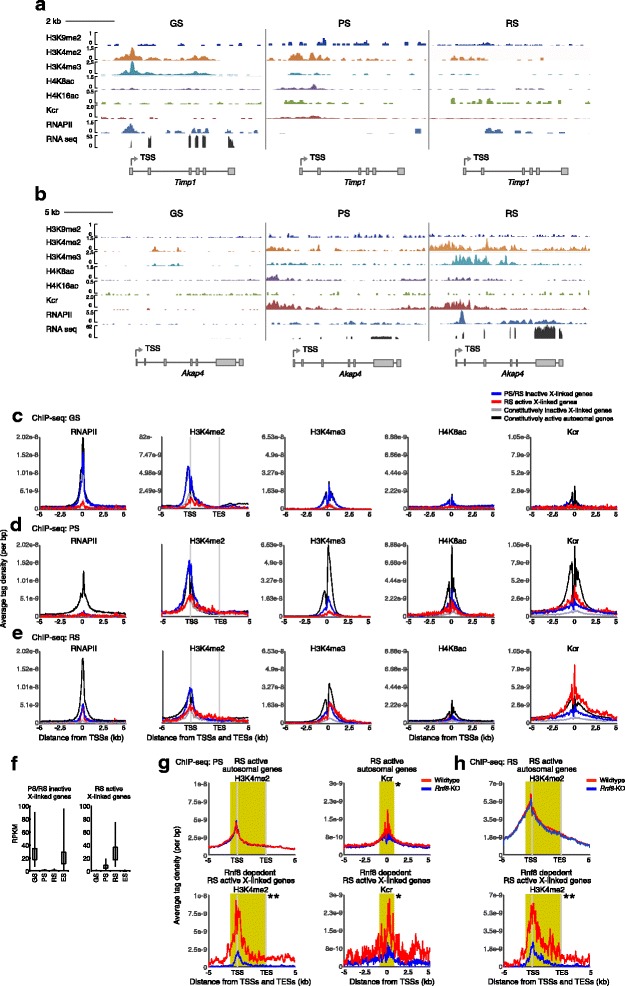
Epigenetic profile of the X chromosome during the late stages of the male germline. **a** Binding peaks of histone marks on PS/RS inactive *Timp1* gene locus in GS, PS, and RS. **b** Binding peaks of histone marks on RS active *Akap4* gene locus in GS, PS, and RS. ATD of histone marks in representative groups in **c** GS, **d** PS, and **e** RS. For H3K4me2, ATD on gene bodies from TSS to TES and ±5 kb regions were analyzed. **f** Average RPKM of each group in GS, PS, RS, and ES. **g**, **h** ATD profiles of H3K4me2 on gene bodies and Kcr on TSS are compared between WT and *Rnf8* KO. Wilcoxon rank sum test was performed for read counts in the highlighted area (H3K4me2: −1 kb from TSS to TES; Kcr: −500 bp to +500 bp from TSS, *P <0.05, **P <0.001). ATD, average tag density; ES, embryonic stem cells; GS, germline stem cells; Kcr, lysine crotonylation; KO, knockout; PS, pachytene spermatocytes; RS, round spermatids; RPKM, reads per kilobase per million; TES, transcription end site; TSS, transcription start site; WT, wild-type

First, we examined the developmental changes in the epigenetic landscape of representative genes from each group during spermatogenesis. The PS/RS inactive gene *Timp1* was marked with H3K4me2 and H3K4me3 in GS, where it is transcribed (Fig. 6a). Upon entry into meiosis, *Timp1* was silenced by MSCI, RNAPII disappeared from the TSS, and the H3K9me2 level increased. However, H3K4me2 and other active modifications remained on the *Timp1* locus, albeit at a lower level in PS. In agreement with the *Timp1* locus, H3K4me2 remained near the TSS of PS/RS inactive X-linked genes in PS despite the disappearance of RNAPII (Fig. 6c,d). These results suggest that MSCI and postmeiotic silencing are established without complete removal of active modifications.

In RS, H3K4me2 and H4K16ac are still present on the *Timp1* locus (Fig. 6a). Paradoxically, the H3K4me2 level at PS/RS inactive X-linked genes was higher than that on RS active X-linked genes at the TSS, and PS/RS inactive X-linked genes had levels of RNAPII and H3K4me3 comparable to that of RS active X-linked genes (Fig. 6e). Importantly, this group of genes was highly expressed in ES cells (Fig. 6f). These results suggest that PS/RS inactive X-linked genes are poised for activation after fertilization, as is the case with PS/RS inactive autosomal genes. However, H3K27me3 was largely depleted from the X chromosome in PS and RS (Fig. 2, Additional file 1: Figure S6). Therefore, PS/RS inactive X-linked genes are poised in RS without the formation of typical bivalent domains.

### Distinct epigenetic features underlie RS-specific gene activation on the X chromosome

As described above, we found that PS/RS active autosomal genes are poised in GS cells for activation during meiosis (Fig. 3). Unlike autosomal genes, X-linked genes that are expressed at later stages possess only a low level of active modifications in GS cells (Fig. 6c, Additional file 1: Figure S6). This result suggests that the X-linked genes are not poised in GS cells for activation during meiosis, which is in accordance with the existence of MSCI and which supports the notion that autosomes and the X chromosome are distinctly regulated in GS cells prior to entry into meiosis.

Next, we examined changes in the epigenetic landscape of RS active X-linked genes during spermatogenesis. On the RS active X-linked gene *Akap4,* which regulates sperm motility, active epigenetic modifications were largely absent in GS cells (Fig. 6b). Upon entry into meiosis, active modifications started to accumulate broadly on the *Akap4* locus with the induction of modest transcription. In RS, H3K4me3, Kcr, and RNAPII were highly accumulated around the TSS, and *Akap4* was robustly expressed. Consistent with this, in ATD analysis, Kcr started to accumulate on the TSSs of RS active X-linked genes in PS (Fig. 6d), and reached a higher level in RS (Fig. 6e). H3K4me2 became enriched on the gene bodies of RS active X-linked genes (Fig. 6e), and RS active X-linked genes were not expressed in ES cells (Fig. 6f). Unlike RS active autosomal genes (Fig. 5c), RS active X-linked genes did not gain a high level of H4K8ac accumulation (Fig. 6e). Therefore, H4K8ac is specifically associated with RS active autosomal genes.

A unique feature of the RS active X-linked genes is that this group of genes escapes postmeiotic silencing of the sex chromosomes. To determine how this group can escape the chromosome-wide silencing of the sex chromosomes, we investigated the profiles of H3K9me2 on the X chromosome. H3K9me2 was consistently high in both groups of X-linked genes compared to autosomal genes and did not exhibit a difference between active and inactive genes in RS, whereas H3K27me3 levels were low (Additional file 1: Figure S6). This result suggests that RS active X-linked gene escape is activated from silent X chromosomes without removing H3K9me2 and instead depends on unique profiles of active modifications.

Previously, we have shown that RNF8 is required for the activation of a subset of escape genes from postmeiotic silencing [21]. To elucidate the regulatory mechanism underlying expression of RS active X-linked genes, we examined how unique profiles of active modifications are established on the X chromosome using the testes of *Rnf8* knockout (KO) mice. Both H3K4me2 and Kcr accumulate on gene bodies and TSSs of RNF8-dependent escape genes (identified in [21]) in an RNF8-dependent manner in PS and RS (Fig. 6g,h). These results further support the conclusion that the unique localization of H3K4me2 and Kcr is important for RS-specific gene activation from the X chromosome.

## Discussion

The bivalent domains we found on somatic/progenitor genes in this study are distinct from those found in previous studies. Because the mission of the germ cells is to maintain the capability to acquire totipotency after fertilization, bivalent domains could help to recover the somatic/progenitor program immediately after fertilization. Therefore, bivalent domains are not limited to developmental promoters, but could be a key feature in soma-to-germ transition. At the same time, late spermatogenesis genes are activated in the late stages of the germline but are inactivated after fertilization. Kcr and H4K8ac, which are enriched on this group of genes, could ensure temporal activation of late spermatogenesis genes without disturbing the germline potential.

Because silencing machinery during meiosis recognizes the sex chromosomes based on their unsynapsed status rather than their gene content [16, 31], it remained unclear whether the sex chromosomes are treated distinctly from autosomes prior to meiosis. Our data are in support of differential regulation prior to meiosis. Consistent with our findings, a recent study demonstrated that duplicated regions that are enriched on the X chromosome are hypomethylated and enriched with H3K9me2 in the germline prior to meiosis [32]. It could be intriguing to further investigate the details about inherent differences between the X chromosome and autosomes in the germline.

Additionally, our analyses establish the epigenomic features of sex chromosome inactivation in the germline. In spite of gene silencing, active modifications are not completely removed from X-linked genes, and most X-linked genes are poised for activation after fertilization. Furthermore, H3K9me2, but not H3K27me3, accumulates ubiquitously on the X chromosomes, and escape from postmeiotic silencing does not involve the removal of H3K9me2 but instead depends on the establishment of active marks downstream of RNF8. These modes of silencing and escape gene activation are very different from those in female X chromosome inactivation. In females, X-linked genes are not poised for later activation, and silent marks such as Xist RNA and H3K27me3 are removed only when escape genes are expressed [33]. Therefore, our analyses clarified a distinct mode of chromosome-wide regulation between males and females.

Taken together, our studies revealed the distinct features of both autosomes and the X chromosome in the male germline. These epigenetic differences could be the basis of distinct evolutionary forces between autosomes and sex chromosomes. In spite of postmeiotic silencing, RS active genes are enriched on the X chromosome (Fig. 1f). This is consistent with Rice’s hypothesis and its supporting reports that male-biased genes are enriched on the X chromosome due to hemizygosity in males, despite the silent environment of the X chromosome in RS in mammals [20, 34, 35]. However, in other species such as *Drosophila*, male-biased genes are actually enriched on the autosomes and not on the X chromosome [36–38]. Because epigenetic events on the sex chromosomes impact their genetic evolution [20], it would be promising to investigate the links between the epigenetic principles we found in this study and their genomic features and evolutionary traits.

## Conclusions

**Fig. 7:**
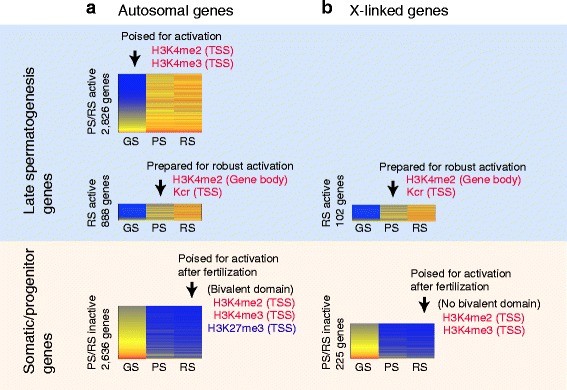
Summary: programmed gene poising for the male germline transcriptomes. See the Discussion section for detail

In this study, our RNA-seq analyses revealed unique features of male germline-specific transcriptomes, and our ChIP-seq analyses further revealed the epigenetic regulation underlying these features. The global gene expression change during the mitosis-to-meiosis transition accompanies both the activation of male reproductive genes and the suppression of somatic/progenitor genes, and these somatic/progenitor genes are re-activated after fertilization to recover the somatic/progenitor program. Importantly, these gene activation events are preprogrammed with poised chromatin during the late stages of the male germline (Fig. 7). First, in GS cells, autosomal PS/RS active genes are poised with H3K4me2 and H3K4me3 at TSSs for activation in PS cells. This result suggests that spermatogenic differentiation is preprogrammed in the stem cell phase of spermatogonia. Second, in PS, RS-specific genes are modified with H3K4me2 and Kcr, thereby preparing the RS transcriptome in PS. We further demonstrate that suppression of somatic/progenitor genes during late spermatogenesis is associated with formation of bivalent domains. A large number of somatic/progenitor genes are poised with bivalent domains and, curiously, H3K27me3 is gradually established on the TSSs of somatic/progenitor genes during the PS to RS transition. Our analyses further clarified the differential regulation between autosomes and sex chromosomes.

## Methods

### Animals

For the wild-type analyses, C57BL/6 mice were used for all experiments. *Rnf8* KO mice were previously reported [39]. All animals were maintained in accordance with the Cincinnati Children’s Hospital Medical Center’s (CCHMC’s) Institutional Animal Care and Use Committee (IACUC) guidelines. All mice were euthanized by cervical dislocation under deep anesthesia or in a CO_2_ gas chamber.

### GS cell culture

GS cells were established from P7 testes and maintained on mitotically inactivated mouse embryonic feeder cells as previously described [40]. For RNA-seq and ChIP-seq analyses, cells were trypsinized, centrifuged at 200 g for 5 min, and then resuspended in GS cell culture medium. Cells were further incubated on a gelatin-coated dish for 30 min to remove mouse embryonic fibroblasts (MEF), and floating cells were collected and used for the experiments.

### Germ cell fractionation

Pachytene spermatocytes and round spermatids were isolated with BSA gravity sedimentation as previously described [41]. Purity was confirmed by nuclear staining with DAPI using fluorescence microscopy. More than 90 % purity was confirmed for each purification.

### ChIP-sequencing

The following primary antibodies were used in this study: RNA polymerase II; CTD clone 8WG16 (05–952; Millipore, Billerica, MA, USA); H3K4me2 (07–030; Millipore); H3K4me3 (9751S; Cell Signaling, Danvers, MA, USA); H3K27me3 (39535; Active Motif, Carlsbad, CA, USA); H3K9me2 (ab1220; Abcam, Cambridge, UK); H4K8ac (07–328; Millipore); H4K16ac (06–762; Millipore); and Kcr (PTM-501; PTM Biolabs, Chicago, IL, USA). For ChIP-seq of histone tail modifications, cells were dissolved in ChIP lysis buffer1 (10 mM Tris–HCl pH 7.6, 150 mM NaCl, 1 mM EDTA, 0.3 % Igepal-630) and incubated at 4 °C for 20 min. After centrifugation at 2,000 rpm for 15 min, the pellet was dissolved in MNase buffer (50 mM Tris–HCl pH 7.6, 3 mM MgCl_2_, 1 mM CaCl_2_, 0.3 M Sucrose, 5 units/mL MNase) at 37 °C for 15 min; the reaction was stopped by adding 1/25 volume of 0.5 M EDTA. Cell lysate was centrifuged at 13,000 rpm for 15 min at 4 °C, and supernatant was collected as chromatin fractions. After adjusting concentrations of NaCl and Igepal-630 to 150 mM and 0.5 %, respectively, the chromatin fractions were incubated with Dynabeads (Life Technologies, Carlsbad, CA, USA) conjugated with antibodies at 4 °C overnight with rotation. Beads were washed three times with wash buffer 1 (50 mM Tris–HCl pH 7.6, 10 mM EDTA, 300 mM NaCl, 0.5 % Igepal-630), two times with wash buffer 2 (50 mM Tris–HCl pH 7.6, 10 mM EDTA, 500 mM NaCl, 0.5 % Igepal-630), and one time with TE buffer containing 250 mM NaCl (TE-250), and incubated in RNase buffer (40 μg/mL RNase A in 500 μL TE-250) at 37 °C for 30 min. Beads were further incubated after adding Proteinase K (100 mg/mL) and 0.5 % SDS at 55 °C for 3 h, and then DNA was collected with the phenol/chloroform and ethanol precipitation method. For ChIP-seq of RNA polymerase II, cross-linking ChIP-seq was performed as described elsewhere [23]. DNA concentration was measured with Qubit 2.0 Fluorometer and Qubit dsDNA HS assay Kit (Invitrogen, Carlsbad, CA, USA). DNA libraries were prepared with NEBNext® ChIP-Seq Library Prep Master Mix Set for Illumina® (NEB, Ipswich, MA, USA) and Agencourt AMPure XP (Beckman Coulter, Brea, CA, USA). DNA libraries were adjusted to 5 nM in TE and sequenced with an Illumina HiSeq 2000. ChIP-seq data were deposited to Gene Expression Omnibus (GEO) under accession number [GSE69946] except input DNA of GS and PS (separately deposited to [GSE55060]). ChIP-seq for H3K4me3, RNAPII, and Kcr in WT, PS, and RS were downloaded (accession numbers: [SRX336649], [SRX336652], [GSM1104368], [GSM1104372], [GSM810675], and [GSM810678]) [11, 42, 43].

### Analysis of ChIP-sequencing results

Data analysis was performed in BioWardrobe Experiment Management System [44, 45]. Briefly, reads were aligned to the mouse genome (mm10) with Bowtie (version 1.0.0 [46]) and fragment length was estimated with MACS [47] and displayed on a local mirror of the UCSC genome browser as coverage by estimated fragments. Heatmap: tags were shifted by ½ of estimated fragment length and tag densities were calculated in 200 bp windows around the TSSs. Average tag density profiles were calculated around TSSs after shifting tags. Resulting graphs were smoothed in 200 bp windows.

### RNA-sequencing

Total RNA was purified using an RNeasy Mini Kit (Qiagen, Limburg, Netherlands) according to the manual. Genomic DNA was removed by column DNase treatment and RNA was eluted with 30 μL of water. RNA quality and quantity were checked by Bioanalyzer (Agilent Technologies, Santa Clara, CA, USA) and Qubit (Life Technologies), respectively. RNA-seq data for ES, priSG-A, SG-A, SG-B, MEF, cerebellum, liver, heart, bone marrow, and spleen were downloaded from GEO (accession numbers: [GSM723776], [GSM723775], [GSM723768], [GSM723772], [GSM723770], [GSM723767], and [GSM723774]). Data analysis was performed in BioWardrobe Experiment Management System [44, 45]. Briefly, reads were mapped to the mm10 genome using TopHat (version 2.0.9 [48]) and assigned to RefSeq-based TSSs (or isoforms) using the BioWardrobe algorithm. Subsequently, enriched gene ontology categories were identified using DAVID v6.7 [49]. Overall, 1,044 autosomal genes were classified to be constitutively expressed (RPKM >5 at GS, PS, and RS, less than two-fold change among GS, PS, and RS), and 8,910 autosomal genes and 500 X-linked genes were constitutively inactivated in all three cell types (RPKM <3 at GS, PS, and RS).

### Data access

RNA-seq and a part of the ChIP-seq data (input DNA of GS and PS) have been submitted to the NCBI GEO [50] under accession number [GSE55060] and have also been used for a parallel study [23]; all other ChIP-seq data have been submitted to the NCBI GEO under accession number [GSE69946].

## Additional file

Additional file 1: Figure S1. RS active genes are specifically expressed in the germline (Supplement to Fig. 1). Expression heat map of 994 autosomal RS active genes at each stage are shown. **Figure S2.** Distinct regulation between the X chromosome and autosomes during the late stages of the male germline (Supplement to Fig. 2). Average tag density (ATD) of epigenetic marks was compared between all autosomal genes and all X-linked genes. (A) GS, (B) PS, and (C) RS are shown. **Figure S3.** Autosomal male reproduction genes are poised in GS cells for activation at PS (Supplement to Fig. 3). (A) ATD of active marks in representative groups in GS cells. (B) ATD of active marks at the genes activated in later stages. **Figure S4.** Active marks remain after the inactivation of autosomal somatic genes in PS (Supplement to Fig. 4). (A) ATD of active marks at the active genes in PS. (B) ATD of active marks at the silent genes in PS. (C) ATD of active marks in PS. **Figure S5.** Epigenetic profiles for RS-specific activation and gene poising on autosomal somatic genes in RS (Supplement to Fig. 5). (A) ATD of H4K16ac at the active genes in RS. (B) ATD of H4K16ac at the silent genes in RS. **Figure S6.** Epigenetic profile of the X chromosome during the late stages of the male germline (Supplement to Fig. 6). (A) ATD of histone marks in representative groups in GS. In contrast to autosomal genes, there is no sign of gene poising on X-linked genes. (B-D) ATD of histone marks in representative groups in GS (B), PS (C), and RS (D). While H3K9me2 is ubiquitously high at both groups of X-linked genes after PS, H3K27me3 and H4K16ac are largely absent from X-linked genes.

## Abbreviations

ATD: average tag density
BSA: bovine serum albumin
ChIP-seq: chromatin immunoprecipitation sequencing
DDR: DNA damage response
ES: embryonic stem cells
GEO: Gene Expression Omnibus
GO: gene ontology
GS: germline stem cells
Kcr: lysine crotonylation
KO: knockout
MEF: mouse embryonic fibroblasts
MSCI: meiotic sex chromosome inactivation
PGC: primordial germ cell
PMSC: postmeiotic sex chromatin
PS: pachytene spermatocytes
RNAPII: RNA polymerase II
RPKM: reads per kilobase per million
RS: round spermatids
TES: transcription end site
TSS: transcription start site
WT: wild-type
