# Supplementary material for: Poised chromatin and bivalent domains facilitate the mitosis-to-meiosis transition in the male germline
Source: BMC Biol. 2015 Jul 22;13:53. doi: 10.1186/s12915-015-0159-8 (PMC4508805; doi:10.1186/s12915-015-0159-8)
Supplement: Additional file 1: Figure S1. — RS active genes are specifically expressed in the germline (Supplement to Fig. 1). Expression heat map of 994 autosomal RS active genes at each stage are shown. Figure S2. Distinct regulation between the X chromosome and autosomes during the late stages of the male germline (Supplement to Fig. 2). Average tag density (ATD) of epigenetic marks was compared between all autosomal genes and all X-linked genes. (A) GS, (B) PS, and (C) RS are shown. Figure S3. Autosomal male reproduction genes are poised in GS cells for activation at PS (Supplement to Fig. 3). (A) ATD of active marks in representative groups in GS cells. (B) ATD of active marks at the genes activated in later stages. Figure S4. Active marks remain after the inactivation of autosomal somatic genes in PS (Supplement to Fig. 4). (A) ATD of active marks at the active genes in PS. (B) ATD of active marks at the silent genes in PS. (C) ATD of active marks in PS. Figure S5. Epigenetic profiles for RS-specific activation and gene poising on autosomal somatic genes in RS (Supplement to Fig. 5). (A) ATD of H4K16ac at the active genes in RS. (B) ATD of H4K16ac at the silent genes in RS. Figure S6. Epigenetic profile of the X chromosome during the late stages of the male germline (Supplement to Fig. 6). (A) ATD of histone marks in representative groups in GS. In contrast to autosomal genes, there is no sign of gene poising on X-linked genes. (B-D) ATD of histone marks in representative groups in GS (B), PS (C), and RS (D). While H3K9me2 is ubiquitously high at both groups of X-linked genes after PS, H3K27me3 and H4K16ac are largely absent from X-linked genes. [file 12915_2015_159_MOESM1_ESM.pdf]

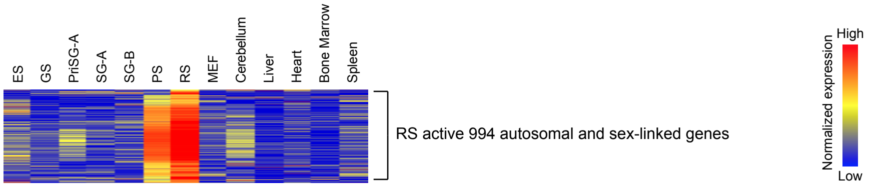

Figure S1. RS active genes are specifically expressed in the germline (Supplement to Figure 1). Expression heat map of 994 autosomal RS active genes at each stage are shown.

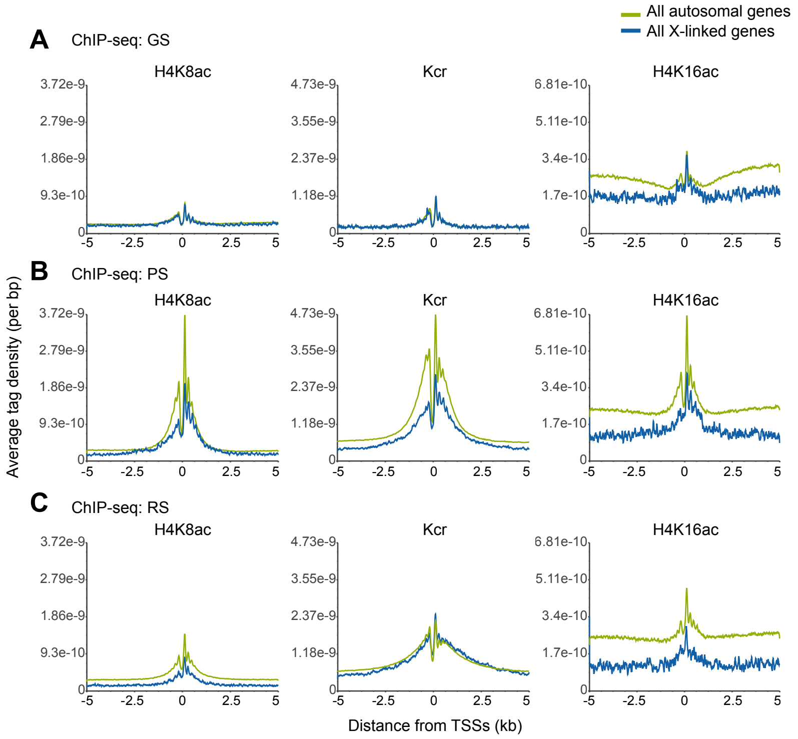

Figure S2. Distinct regulation between the X chromosome and autosomes during the late stages of the male germline (Supplement to Figure 2). Average tag density (ATD) of epigenetic marks was compared between all autosomal genes and all X-linked genes. (A) GS, (B) PS, and (C) RS are shown.

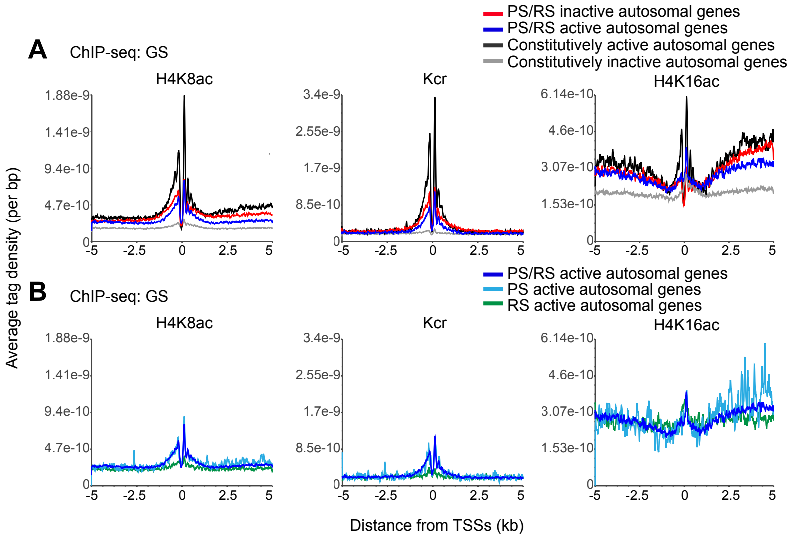

Figure S3. Autosomal male reproduction genes are poised in GS cells for activation at PS (Supplement to Figure 3). (A) ATD of active marks in representative groups in GS cells. (B) ATD of active marks at the genes activated in later stages.

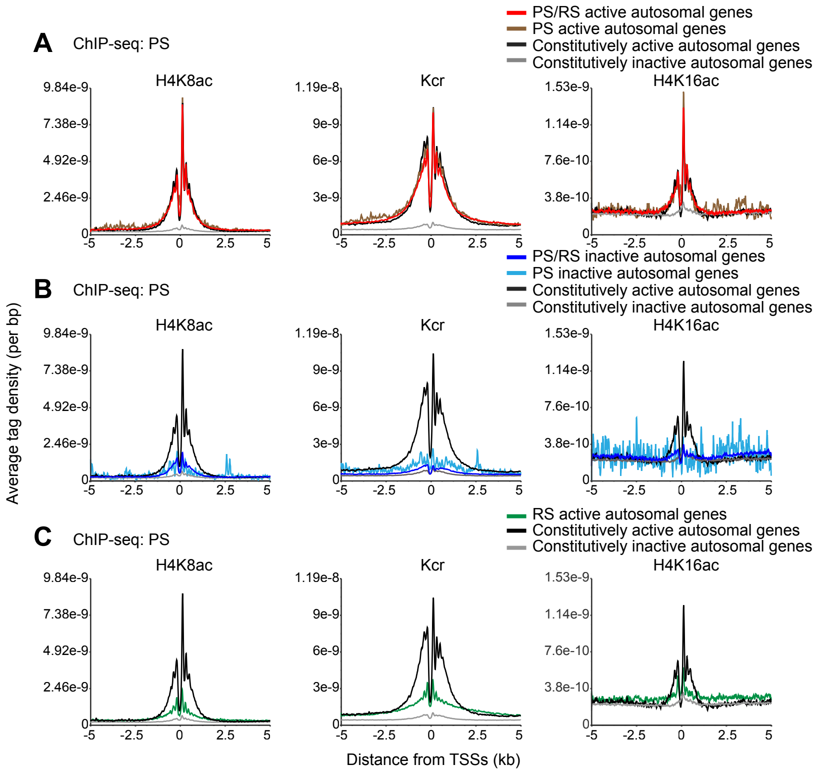

Figure S4. Active marks remain after the inactivation of autosomal somatic genes in PS (Supplement to Figure 4). (A) ATD of active marks at the active genes in PS. (B) ATD of active marks at the silent genes in PS. (C) ATD of active marks in PS.

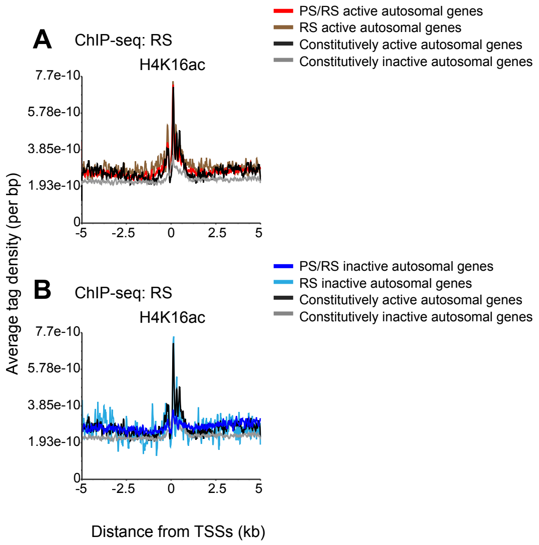

Figure S5. Epigenetic profiles for RS-specific activation and gene poising on autosomal somatic genes in RS (Supplement to Figure 5). (A) ATD of H4K16ac at the active genes in RS. (B) ATD of H4K16ac at the silent genes in RS.

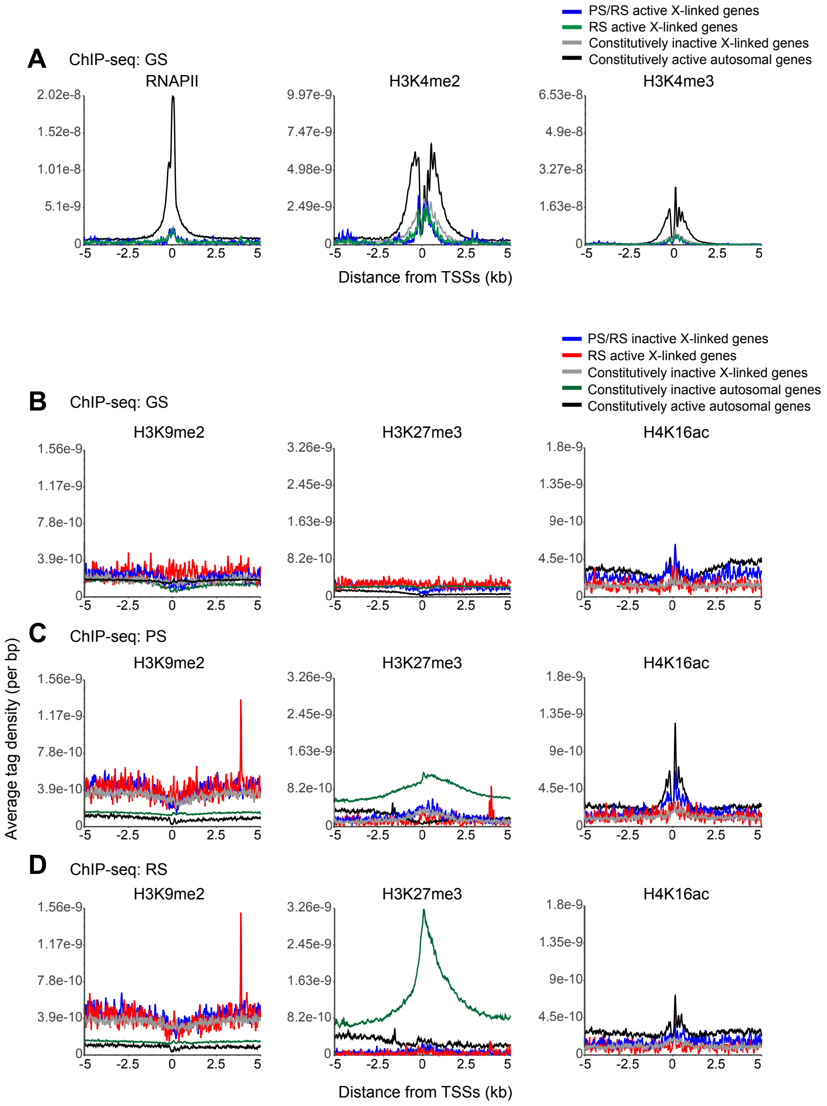

Figure S6. Epigenetic profile of the X chromosome during the late stages of the male germline (Supplement to Figure 6). (A) ATD of histone marks in representative groups in GS. In contrast to autosomal genes, there is no sign of gene poising on X-linked genes. (B-D) ATD of histone marks in representative groups in GS (B), PS (C), and RS (D). While H3K9me2 is ubiquitously high at both groups of X-linked genes after PS, H3K27me3 and H4K16ac are largely absent from X-linked genes.
